# Heavy Metal-Induced Expression of PcaA Provides Cadmium Tolerance to *Aspergillus fumigatus* and Supports Its Virulence in the *Galleria mellonella* Model

**DOI:** 10.3389/fmicb.2018.00744

**Published:** 2018-04-13

**Authors:** Fruzsina Bakti, Christoph Sasse, Thorsten Heinekamp, István Pócsi, Gerhard H. Braus

**Affiliations:** ^1^Institute for Microbiology and Genetics, Department of Molecular Microbiology and Genetics, Goettingen Center for Molecular Biosciences, University of Göttingen, Göttingen, Germany; ^2^Department of Biotechnology and Microbiology, Faculty of Science and Technology, University of Debrecen, Debrecen, Hungary; ^3^Department of Molecular and Applied Microbiology, Leibniz Institute for Natural Product Research and Infection Biology, Hans Knöll Institute, Jena, Germany

**Keywords:** PcaA, *Aspergillus fumigatus*, ATPase, cadmium tolerance, copper, virulence factor, *Galleria mellonella*

## Abstract

Most of the metal transporters in *Aspergillus fumigatus* are yet uncharacterized. Their role in fungal metabolism and virulence remains unclear. This paper describes the novel PIB-type cation ATPase PcaA, which links metal homeostasis and heavy metal tolerance in the opportunistic human pathogen *A. fumigatus*. The protein possesses conserved ATPase motif and shares 51% amino acid sequence identity with the *Saccharomyces cerevisiae* cadmium exporter Pca1p. A *pcaA* deletion, an overexpression and a *gfp-pcaA* complementation strain of *A. fumigatus* were constructed and their heavy metal susceptibilities were studied. The *pcaA* knock out strain showed drastically decreased cadmium tolerance, however, its growth was not affected by the exposure to high concentrations of copper, iron, zinc, or silver ions. Although the lack of PcaA had no effect on copper adaption, we demonstrated that not only cadmium but also copper ions are able to induce the transcription of *pcaA* in *A. fumigatus* wild type Af293. Similarly, cadmium and copper ions could induce the copper exporting ATPase *crpA*. These data imply a general response on the transcriptomic level to heavy metals in *A. fumigatus* through the induction of detoxification systems. Confocal microscopy of the *gfp-pcaA* complementation strain expressing functional GFP-PcaA supports the predicted membrane localization of PcaA. The GFP-PcaA fusion protein is located in the plasma membrane of *A. fumigatus* in the presence of cadmium ions. Virulence assays support a function of PcaA for virulence of *A. fumigatus* in the *Galleria mellonella* wax moth larvae model, which might be linked to the elimination of reactive oxygen species.

## Introduction

*Aspergillus fumigatus* is a competitive saprophytic mold widespread in nature, mostly found in soil and on decaying organic matter. Its airborne conidiospores are inhaled into our lungs and neutrophils trigger programmed cell death with apoptosis-like features (Shlezinger et al., 2017). Immune suppressed individuals are susceptible to the fungus, which can cause invasive aspergillosis (Tekaia and Latgé, 2005). As a saprophyte as well as a pathogen, *A. fumigatus* has the ability for rapid adaptation to changing environmental conditions, may it be heavy metal pollution or oxidative stress exerted by the host cell as part of its defense mechanism (Aguirre et al., 2006). This ability manifests in a wide tolerance of this fungus for unfavorable conditions such as heat (Jesenská et al., 1993; Bhabhra and Askew, 2005), heavy metal (Fazli et al., 2015), oxidative stress (Qiao et al., 2008; Muszkieta et al., 2014) or resistance against antifungal agents (Wiederhold and Patterson, 2015; Brown and Goldman, 2016).

Metal ions such as iron, zinc, and copper fulfill fundamental cellular functions in trace amounts, others, like cadmium or silver ions are dispensable for life or even toxic. Iron, among other functions, takes part in vital biological mechanisms, like cellular respiration as a component of the electron transport chain. Copper and zinc ions are incorporated into essential enzymes. Such an enzyme is the Cu/Zn superoxide dismutase (Cu/Zn SOD) with a crucial role in elimination of reactive oxygen species (ROS), therefore, it contributes to the virulence of *Candida albicans* (Hwang et al., 2017). Laccases, involved in melanin biosynthesis and in virulence of *A. fumigatus*, also bind copper as cofactor (Upadhyay et al., 2013). Cadmium on the other hand, is an environmental pollutant which causes the break of the redox-balance and inactivates DNA repair even in low concentrations (McMurray and Tainer, 2003; Bertin and Averbeck, 2006). Noteworthy, high concentrations of copper ions have similar effects to those of cadmium in low concentrations: copper excess leads to the perturbation of the redox status of the cell (Gaetke and Chow, 2003). This biocidal property of copper made it a long-used antimicrobial, antifungal agent (Borkow and Gabbay, 2009). Cells of the innate immune response, the macrophages also use copper ions to eliminate pathogens (Festa and Thiele, 2011; Stafford et al., 2013; Ding et al., 2014; Djoko et al., 2015).

The balance of the metal homeostasis and virulence requires the fine-tuning of metal translocating proteins in pathogenic species by either import, export, or intracellular exchange of metal ions (Festa and Thiele, 2012; Waterman et al., 2012; Ding et al., 2014; Zhang et al., 2016). Export of metal ions (such as Cu^2+^, Ag^+^, Zn^2+^, and Cd^2+^) typically takes place through PIB-type ATPases (Kühlbrandt, 2004). Adle et al. (2007) described a metal inducible PIB-type ATPase, Pca1p in *Saccharomyces cerevisiae*. Pca1p is localized to the plasma membrane and provides exceptional cadmium- and elevated copper resistance to the baker’s yeast (Adle et al., 2007). The corresponding ortholog in the dimorphic yeast pathogen *C. albicans* is CRP1 (also known as CRD1) (Riggle and Kumamoto, 2000; Weissman et al., 2000). CRP1 is assigned to the detoxification of copper, cadmium and silver, since the *crp1* null mutant showed extreme sensitivity to these metals (Riggle and Kumamoto, 2000). CRP1 is also required for the full virulence of *C. albicans* (Mackie et al., 2016). An ortholog of CRP1 was found in *A. fumigatus*: a copper exporting ATPase, CrpA (Wiemann et al., 2017).

*Aspergillus fumigatus* is a successful saprophytic filamentous fungus which is able to adjust to various unfavorable environmental conditions. Elevated cadmium resistance of *A. fumigatus* was demonstrated presumably due to the presence of Pca1p-type transporters (De Vries et al., 2017). In our work we focused on describing Pca1p-type proteins which confer cadmium tolerance to *A. fumigatus*.

## Materials and Methods

### Strains, Medium, Growth Conditions

The DH5α and DH10B (Invitrogen) strains of *Escherichia coli* used for cloning were grown in LB-medium (1% tryptone, 0.5% yeast extract, 1% NaCl, and 2% agar for solid cultures) at 37°C. For selection 100 mg/ml ampicillin was used. The wild type (WT) Af293 *A. fumigatus* strain served as parental strain for all strains of this study. Selection of the correct clones occurred on medium containing 150 ng/ml pyrithiamine. The *A. fumigatus* strains used for this paper (Supplementary Table S1) were maintained on aspergillus nitrate minimal medium (Pontecorvo et al., 1953). The experiments were carried out in modified minimal medium [1% D-glucose; 1x Aspergillus salt solution (7 mM KCl, 4.3 mM MgSO_4_, 11.2 mM KH_2_PO_4_); 10 mM NaNO_3_; 1x trace elements (7.1 μM CoCl_2_, 6.4 μM CuSO_4_, 174 μM EDTA, 18 μM FeSO_4_, 178 μM H_3_BO_3_, 6.2 μM Na_2_MoO_4_, 25 μM MnCl_2_, 76 μM ZnSO_4_ pH 6.5); pH 6.5 (Käfer, 1977)]. For the surface cultures, medium was supplemented with 2% agar. All *A. fumigatus* strains were grown at 37°C. Freshly grown 3 days old conidiospores were suspended in saline-tween solution (0.96% NaCl-0.02% Tween 20) and used for the experiments.

### Plasmid Constructions

All primers and plasmids used in this study are listed in Supplementary Tables S3, S4, respectively. DNA fragments for plasmid constructions were amplified from *A. fumigatus* WT Af293 genomic DNA. For plasmids pFB03 and pFB22 the pBluescript II KS+ restriction digested by *EcoR*V served as backbone and the recyclable cassette with pyrithiamine was used as marker (Hartmann et al., 2010). The 5′ and the 3′ flanking regions of *pcaA* were amplified using the primer pair pca1-1/pca1-2 and pca1-3/pca1-4, respectively, to construct the deletion plasmid pFB03. For pFB22, the 5′ flanking region was amplified using the primers FB075/FB033. The GFP with an N-terminal linker sequence was amplified from the plasmid pME4292 (Jöhnk et al., 2016) with the primers SR120/SR121. The coding sequence of *pcaA* with overhang to the GFP was amplified with FB034/FB078. The 3′ flanking region was obtained by PCR amplification with the primers FB077/FB084. The deletion- and the *gfp-pcaA* complementation cassettes were obtained by restriction digestion of the plasmid pFB03 and pFB22 by *Mss*I, respectively. Ectopic integration of the pFB08 into *A. fumigatus* Af293 WT resulted in the overexpression strain of *pcaA* (OE *pcaA*). To construct pFB08, the coding sequence of *pcaA* was amplified (using primers FB009/FB008) and cloned into the *Mss*I site of pSK379 (Wagener et al., 2008), where the expression of *pcaA* is driven by the constitutive *gpdA* promoter. We used the methods described by Inoue et al. (1990) and Punt and van den Hondel (1992) for transformation of *E. coli* and *A. fumigatus*, respectively. Southern hybridization analyses were performed to verify the correct insertion of the constructs for Δ*pcaA*, *gfp-pcaA* and the OE *pcaA* strains (Supplementary Figures S1A–C). Besides the Southern experiments, the *pcaA* gene expression was analyzed by qRT-PCR in the OE *pcaA* strain, together with the Δ*pcaA* and the *gfp-pcaA* strains (Supplementary Figure S2).

### Plate Assays

The phenotypical analyses were carried out on solid modified minimal medium supplemented with different concentrations of CdSO_4_, CuSO_4_, Fe_2_SO_4_, ZnCl_2_, AgNO_3_, and menadione sodium bisulfite (MSB). 3000 conidiospores/strain were point inoculated on agar plates and grown for 3 days at 37°C.

### Gene Expression Measurements

Transcription of *pcaA* and *crpA* was analyzed by qRT-PCR with the primers FB094/FB095 and FB104/FB105, respectively. As housekeeping gene the histone *h2A* was used and amplified with the primers KT316 and KT317. For the RNA extraction 5 × 10^6^ conidiospores/ml/strain were inoculated in 100 ml modified minimal medium and shaken for 20 h, then supplemented with 300 μM CdSO_4_ or 300 μM CuSO_4_, or 20 mM Fe_2_SO_4_, or 20 mM ZnCl_2_. Samples were taken at different time points from all cultures. Cultures without supplementation were used as control. Mycelia were collected at the indicated time points, washed with saline and pulverized for RNA isolation. Total RNA of the samples was extracted with the “RNeasy plant mini kit” (QIAGEN). 0.8 μg RNA was used as template for cDNA synthesis by the QuantiTect Reverse Transcription Kit (QIAGEN). For this analysis the CFX Connect^TM^ Real Time System (Bio-Rad) cycler was applied. Mesa Green qPCR^TM^ MasterMix Plus for SYBR^®^ assay with Fluorescein (Eurogentec) was used as a fluorophore for the measurements. The gene expression analyses were performed in 2–4 independent biological experiments. Every independent experiment was performed in three technical replicates. The data were analyzed by the CFX Manager^TM^ Software version 3.1 (Bio-Rad). Two-sided *t*-test was used as statistical test to determine significance with twofold taken as regulation threshold. Transcription of the gene of interest was quantified relative to the *h2A* in ΔΔCT method (Livak and Schmittgen, 2001).

### Protein Extraction and Immunoblotting

For protein extraction 5 × 10^6^ conidiospores/ml of the *gfp-pcaA* strain were inoculated in 100 ml modified minimal medium and shaken for 20 h, then supplemented with 300 μM CdSO_4_. Samples were taken at the different time points from all cultures. Cultures without supplementation were used as control. Mycelia were collected at the indicated time points, washed with saline (containing 100 μM PMSF and 0.1% DMSO) and pulverized for protein extraction. The ground mycelium was re-suspended with 4 M urea-buffer B^∗^ [100 mM Tris-HCl pH 7.5, 300 mM NaCl, 10% glycerol, 2 mM EDTA pH 8.0, 0.02% NP-40, freshly supplemented with 2 mM DTT and cOmplete^TM^ Protease Inhibitor Cocktail (ROCHE)]. After the centrifugation, the pellet fraction -containing membrane proteins- was re-suspended in 4 M urea. The samples from the pellet fraction were boiled at 95°C for 5 min then used for analyses. The protein concentrations were determined by NanoDrop^TM^ (Thermo Scientific). Equal amounts of protein were loaded on 12% SDS gels and transferred onto nitrocellulose membrane. The PcaA protein induction pattern was followed by a functional GFP-PcaA fusion protein. Membranes were hybridized with mouse monoclonal α-GFP antibody (sc-9996, Santa Cruz) and re-probed with α- tubulin antibody (T0926, Sigma-Aldrich) for loading control.

### Confocal Microscopy

Fluorescence microscopy was performed with the Zeiss Observer Z.1 microscope. 500 conidiospores of the *gfp-pcaA* strain was inoculated and grown in eight well microscopy chambers (Ibidi^®^) containing 400 μl liquid medium for 20 h at 37°C. After 20 h the medium was supplemented with 300 μM CdSO_4_, 300 μM CuSO_4_, 20 mM ZnCl_2_, or 20 mM Fe_2_SO_4_. Cultures without supplementation served as controls for this experiment. Pictures were made by the SlideBook 6.0 software (Intelligent Imaging Innovations) in the time interval from 30 to 180 min after supplementation.

### Virulence Test

The virulence of the Δ*pcaA*, OE *pcaA* strain, *gfp-pcaA* complementation and the WT strain were compared in the *Galleria mellonella* infection model. Three biological experiments were performed to analyze virulence. For individual experiments groups of 12–28 larvae per *A. fumigatus* strain were used. Suspension of 8 × 10^4^ conidiospores in 20 μl saline was injected into the hemocoel via the last right proleg of the larvae. As controls “untreated” (mock) larvae and “saline control” larvae, injected with 20 μl saline solution, were co-incubated. The larvae were incubated at 37°C for 7 days. The survival of the larvae was monitored daily after the infection. The saline solution contained 0.5 mg/ml rifampicin for the virulence test (Slater et al., 2011; Gomez-Lopez et al., 2014). GraphPad Prism 7.00 software for Windows (GraphPad Software, La Jolla, CA, United States) was used for the analysis of the virulence data set. The survival curves were compared with the log-rank (Mantel–Cox) test. Differences between the experimental groups were considered statistically significant with *P*-values below 0.0001.

## Results

### PcaA Carries the Features of a PIB-Type ATPase

Sequence homology search was performed against Pca1p (UniProt ID: P38360) to identify Pca1p-like proteins in the genome of *A. fumigatus*. BLASTp screening for Pca1p-type proteins revealed high sequence identity (51%) with the deduced amino acid sequence of Afu1g16130. Accordingly, Afu1g16130 was named PcaA for P-type cation-transporting ATPase. Interspecies comparison with the full length deduced amino acid sequence of PcaA explores similarities with further PIB-type transporters, such as the copper-exporter CRP1 of *C. albicans* (Mackie et al., 2016; identity 28%), the copper importer CtpA (Upadhyay et al., 2013; identity 29.6%) and the copper exporter CrpA (Wiemann et al., 2017; identity 26.4%) of *A. fumigatus*. The alignment of the Heavy Metal Associated (HMA; InterPro ID: IPR006121) domains of these proteins supports their common origin (Supplementary Table S2).

**FIGURE 1:**
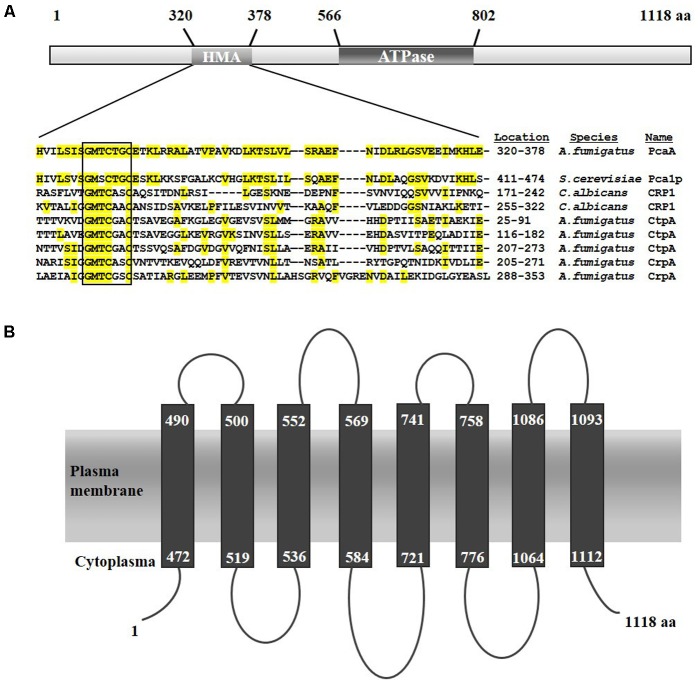
Schematic predicted structure of the *Aspergillus fumigatus* PcaA. **(A)** The conserved HMA domains of the related metal transporting proteins are presented (according to Uniprot). Identical amino acid residues in the HMA domain of *A. fumigatus* PcaA, *Saccharomyces cerevisiae* Pca1p, *Candida albicans* CRP1, *A. fumigatus* CtpA and CrpA are highlighted in yellow. PcaA and Pca1p bear a single HMA domain, whereas CRP1, CtpA and the putative copper transporter CrpA possess more than one HMA domain. **(B)** Model of the eight putative transmembrane segments of PcaA according to Phyre2 prediction (MEMSAT-SVM method).

*In silico* analysis of the deduced protein sequence of PcaA revealed a HMA domain carrying the conserved GMXCXXC motif (shown in box, **Figure 1A**). Moreover, a PIB-type ATPase domain is spanning from the amino acid residue 566 to 802 (InterPro ID: IPR027256) with the conserved CPC (position 771) and DKTGT (position 815) motif. Sequence homology modeling of PcaA executed by Phyre2 (Kelley et al., 2015) discovered eight membrane spanning helices which are typical for PIB-type ATPases (Kühlbrandt, 2004) (**Figure 1B**).

### PcaA Is Required for Cadmium, but Negligible for Copper, Iron, Zinc, and Silver Tolerance of *A. fumigatus*

**FIGURE 2:**
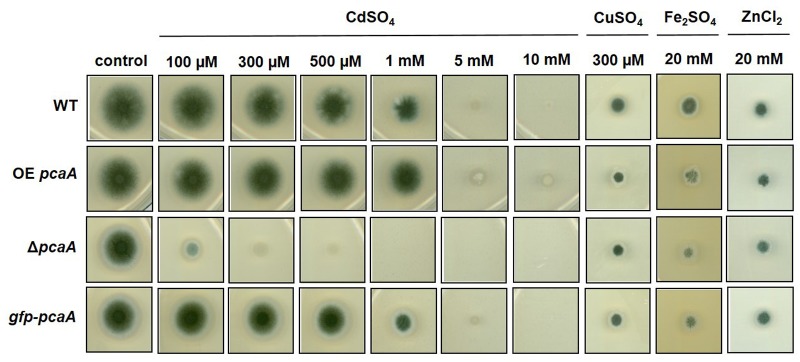
Growth of Δ*pcaA*, OE *pcaA*, and the *gfp-pcaA* strains on metal containing agar plates. Metal susceptibility of the above mentioned strains was tested on agar plates containing concentrations of CdSO_4_ ranging between 100 μM and 10 mM; or 100 μM to 2 mM CuSO_4_, 5 to 25 mM Fe_2_SO_4_ or 5 to 25 mM ZnCl_2_ (Supplementary Figure S3), although here we only present the effect of 300 μM CdSO_4_, 300 μM CuSO_4_, 20 mM Fe_2_SO_4_ and 20 mM ZnCl_2_. These concentrations were used for further experiments as they were sufficient to cause growth defect without complete inhibition. Compared to the wild type (WT), even low concentrations of cadmium strongly hindered the Δ*pcaA* strain, however, this growth defect was restored in the *gfp-pcaA* complementation strain. The OE *pcaA* could propagate colony even on 10 mM cadmium sulfate containing plates. Copper, iron, or zinc ions had no considerable impact on the Δ*pcaA* or the OE *pcaA* strain in comparison to the WT in any of the above mentioned concentrations.

A *pcaA* deletion strain was constructed to analyze the role of PcaA in metal adaptation. The growth of the Δ*pcaA*, *gfp-pcaA* complementation together with a ^P^*gpdA-pcaA* overexpression (OE *pcaA*) strain was tested in the presence of increasing concentrations of CdSO_4_, CuSO_4_, Fe_2_SO_4_, ZnCl_2_ (**Figure 2**) and AgNO_3_ (Supplementary Figure S3). The phenotypical analysis showed drastically increased sensitivity of Δ*pcaA* to cadmium: the growth of the deletion strain was strongly reduced in presence of cadmium ions compared to the WT. The growth defect of Δ*pcaA* strain caused by cadmium ions was restored by the re-introduction of a single copy of *pcaA* into the Δ*pcaA* strain (*gfp-pcaA* complementation strain). In contrast to this, the OE *pcaA* strain achieved elevated resilience resulting in an increased colony size at higher concentrations of cadmium sulfate (**Figure 2**). The sensitivity was specific to cadmium: neither copper nor iron or zinc or silver ions had perceptible impact on the growth of the deletion or overexpression *pcaA* strain in comparison to the WT (**Figure 2** and Supplementary Figure S3). These findings indicate the relevance of PcaA for cadmium detoxification in *A. fumigatus.*

### Cadmium Ions Induce PcaA Protein Formation, Which Accumulates in the Plasma Membrane of *A. fumigatus*

**FIGURE 3:**
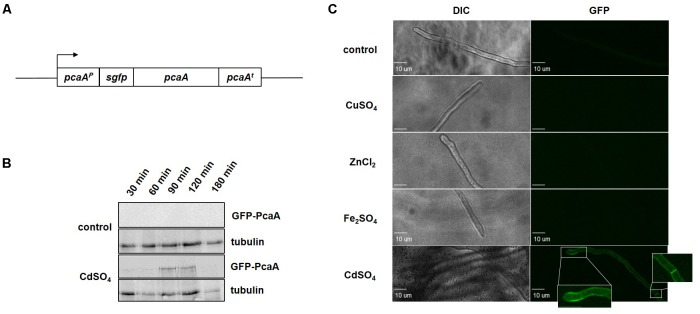
Cadmium ions induce PcaA which is localized in the plasma membrane. **(A)** Schematic representation of the *pcaA* locus of the *gfp-pcaA* strain. Cultures of the *gfp-pcaA* strain, which expresses a functional GFP-PcaA fusion protein were used to visualize PcaA *in vitro* and *in vivo*. **(B)** Immunoblotting was carried out with protein samples deriving from *gfp-pcaA* cultures with 300 μM cadmium sulfate and without supplementation. Samples were taken at the indicated time points following the supplementation. Samples from unstressed cultures served as control respective to each time points. The western hybridization with a monoclonal α-GFP antibody showed the induction of the GFP-PcaA fusion protein from 90 min after cadmium was added, whereas there were no signals in the controls. α-tubulin was used as loading control. **(C)** The subcellular localization of the GFP-PcaA was monitored in the *gfp-pcaA* strain using a confocal microscope. The micrographs were taken in the time interval from 30 to 180 min following the supplementation with 300 μM CuSO_4_, 300 μM CdSO_4_, 20 mM Fe_2_SO_4_ and 20 mM ZnCl_2_. No signals were detected in the control (cultures without supplementation), or when CuSO_4_, Fe_2_SO_4_ or ZnCl_2_ was added to the culture. Accumulation of GFP-PcaA signal in the membrane of the hyphal tip and septae of *gfp-pcaA* strain was visible in the presence of CdSO_4_. Size bar: 10 μm.

In order to examine the effect of cadmium and copper ions for the cellular PcaA levels and to allocate PcaA within the fungal cell, a *gfp-pcaA* strain -expressing a functional GFP-PcaA fusion protein- was constructed and studied (**Figure 3A**). The cellular PcaA level was examined *in vitro* by western hybridization. GFP-PcaA signal could be shown 90 min after cadmium sulfate was added to the culture. Exposure to cadmium resulted in GFP-PcaA induction, whereas without cadmium supplementation (control) no signal was detected by western hybridization (**Figure 3B**). Presence of copper ions did not result in detectable GFP-PcaA levels either (Supplementary Figure S4).

Cultures of *gfp-pcaA* strain supplemented with cadmium sulfate, copper sulfate, iron sulfate, and zinc chloride were monitored by confocal microscopy to determine the cellular localization of PcaA. In good agreement with our *in silico* protein sequence analysis (**Figure 1B**), GFP-PcaA was enriched in the plasma membrane including septal membrane of *A. fumigatus* when cadmium was in the medium (**Figure 3C**). In the presence of copper, iron, and zinc or in the control cultures without supplementation no signal could be observed.

### Both Cadmium and Copper Ions Can Induce the Transcription of the *pcaA* Gene

**FIGURE 4:**
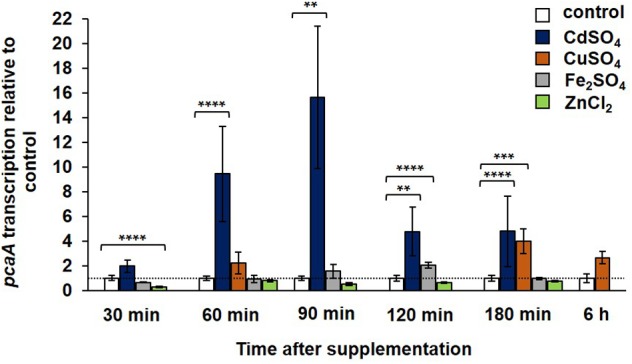
Transcription of *pcaA* in the presence and absence of metals. The *pcaA* transcription was monitored in the WT strain at different time points after supplementation with 300 μM CuSO_4_, 300 μM CdSO_4_, 20 mM Fe_2_SO_4_ and 20 mM ZnCl_2_. Samples from cultures without supplementation were used as control, respective to each timepoint (expression was set to 1). The error bars represent the standard error of the mean (SEM) of three technical replicates in at least two independent measurements. The asterisks indicate the significances between the control and the experimental sample (two-sided *t*-test, ^∗^*P* < 0.05; ^∗∗^*P* < 0.01; ^∗∗∗^*P* < 0.005; ^∗∗∗∗^*P* < 0.001). The dashed line indicates the expression of the control samples.

Gene expression of *pcaA* was studied in the WT strain at different time points following the supplementation with cadmium sulfate, copper sulfate, iron sulfate, and zinc chloride. A divergent expression pattern was observed in the presence of these metals. Approximately after 60 min cadmium exposure the transcription of *pcaA* was significantly, up to 10-fold increased (**Figure 4**). The expression reached its highest peak 90 min after cadmium sulfate supplementation, resulting in approximately 15-fold upregulation of the *pcaA* transcription compared to the control. This correlates to our western hybridization data, where we detected GFP-PcaA signal after 90 min of cadmium exposure (**Figure 3B**). The peak of the expression decreased during prolonged incubation in cadmium containing medium until it reached the expression levels of unstressed cultures. Thus, *pcaA* expression is induced by cadmium in a time-dependent manner. Although surface cultures showed no significant phenotypical differences in presence of 300 μM CuSO_4_ (**Figure 2**), it was still sufficient to trigger the transcription of *pcaA* in liquid cultures of the WT. The impact of copper ions for *pcaA* gene expression was weaker and delayed as the cadmium’s, yet, a fourfold upregulation could be measured 180 min after copper was added to the culture (**Figure 4**). Differently from cadmium and copper, iron, and zinc hardly influenced the *pcaA* expression. Iron slightly induced *pcaA* resulting in approximately twofold upregulation after 120 min exposure, whereas zinc slightly suppressed (nearly 2.5-fold downregulation) the *pcaA* transcription 30 min after supplementation (**Figure 4**).

### *crpA* Transcription Is Upregulated Under Copper and Cadmium Exposure

**FIGURE 5:**
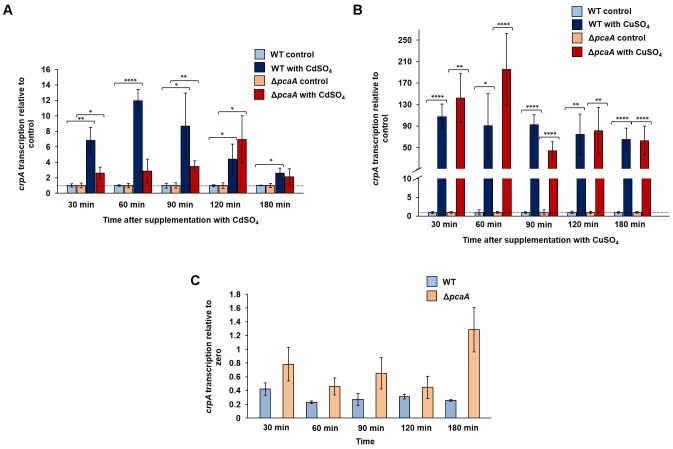
Gene expression of *crpA* in WT and in the Δ*pcaA* strain in the presence or absence of cadmium and copper ions. The *crpA* transcription was monitored at different time points after supplementation with 300 μM CdSO_4_ or 300 μM CuSO_4_. Samples from cultures without supplementation were used as control, respective to each time point (expression was set to 1). The error bars represent the SEM of three technical replicates in two to four independent measurements. The asterisks indicate the significances between the control and the experimental sample (two-sided *t*-test, ^∗^*P* < 0.05; ^∗∗^*P* < 0.01; ^∗∗∗^*P* < 0.005; ^∗∗∗∗^*P* < 0.001). The dashed line indicates the expression of the control samples. **(A)** The *crpA* transcription was upregulated by cadmium ions significantly up to 12-fold (60 min after supplementation) in the WT, but in the lack of PcaA this transcription induction was less pronounced: up to sixfold (120 min after supplementation). **(B)** The supplementation of WT and Δ*pcaA* strain cultures with 300 μM CuSO_4_ resulted in similarly high *crpA* transcription in both strains during the cultivation time (30 to 180 min after supplementation). **(C)** Graph represents the *crpA* transcription in the control cultures (without supplementation) of WT and the Δ*pcaA* strain, where the gene expression is relative to zero.

We examined, whether cadmium ions are able to promote transcription of genes encoding copper transporters, in a *vice versa* control to the observed increased transcription of the cadmium transporter gene *pcaA* by copper ions. The transcription of the copper exporter gene *crpA* was monitored in the WT and in the Δ*pcaA* strain at different time points after cadmium or copper sulfate was added to the culture. A significant 12-fold increase of *crpA* transcription was observed in the WT cultures after 60 min exposure of cadmium compared to the control sample, which was not supplemented (**Figure 5A**). In the Δ*pcaA* strain the *crpA* upregulation was less extent and delayed compared to the WT. It reached its highest sixfold upregulation only 120 min after the supplementation (**Figure 5A**). An intact *pcaA* gene might be involved in the *crpA* expression induction in the presence of toxic cadmium ions. It was analyzed whether PcaA is also involved copper ion dependent *crpA* transcription. The expression of *crpA* was up to 140-times upregulated in the WT cells compared to the control samples. Similarly, high transcript levels of the copper transporter *crpA* were observed in the absence of *pcaA* under exposure to copper ions (**Figure 5B**). There was no significant difference in the *crpA* transcription between the cultures of the wild type and the Δ*pcaA* strain without supplementation in the above mentioned time points (**Figure 5C**).

### AfYap1 Contributes to the Wild Type-Like Cadmium and Copper Tolerance

**FIGURE 6:**
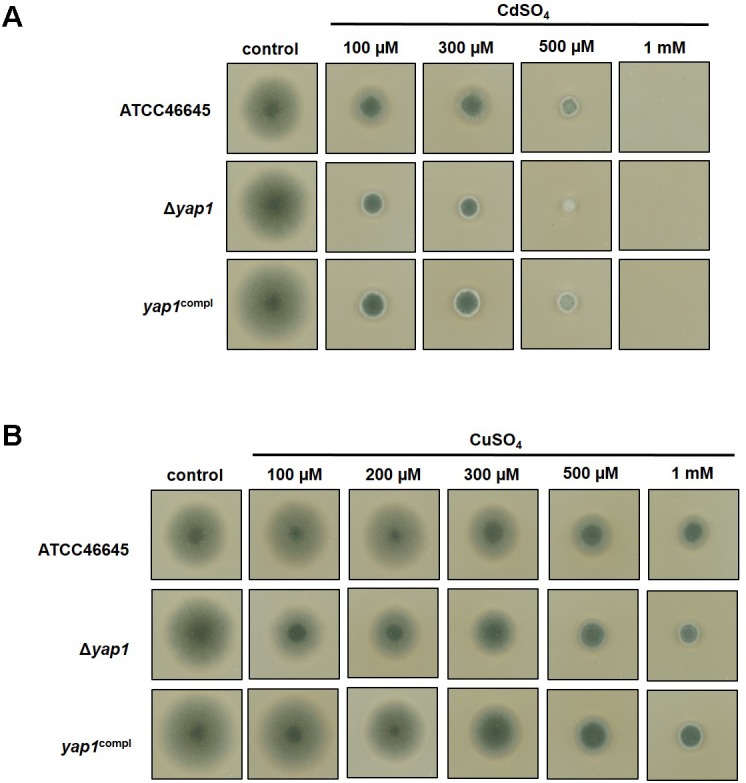
Growth of Δ*yap1* strain of *A. fumigatus* on copper and cadmium containing plates. The growth of the Δ*yap1* strain was followed in the presence of increasing concentrations of CdSO_4_ (in a range of 100 μM to 5 mM) and CuSO_4_ (ranging from 100 μM to 1 mM) in comparison to *yap1*^compl^ strain and the WT ATC45546. **(A)** Cadmium ions have a pronounced negative effect on the growth of the Δ*yap1* strain even at lower concentrations compared to the normal growth conditions (unsupplemented, control plates), whereas the same concentration of cadmium ions have less toxic effect on the WT. **(B)** The presence of increased concentrations of copper ions reduce the growth of the Δ*yap1* strain compared to WT.

We analyzed the role of AfYap1, a possible common regulator for cadmium and copper sensing. AfYap1 is an ortholog of the yeast Yap1 transcription factor for metal sensing, which is conducted through its cysteine- rich cadmium sensing domain located at the C-terminus (Wu et al., 1993; Azevedo et al., 2007). The cadmium and copper susceptibility of the *A. fumigatus* Δ*yap1* strain (Lessing et al., 2007) was investigated. The growth of the Δ*yap1* strain was compared to the ATCC46645 WT or the *yap1*^compl^ strain. The presence of low concentration (100 μM) cadmium sulfate resulted in approximately 66% (± 2%) decrease in colony size of the Δ*yap1* strain compared to the normal growth conditions (control), whereas only 25% (± 10%) cadmium mediated growth inhibition was observed in the ATCC46645 WT strain (**Figure 6A**). Growth defect of the Δ*yap1* strain was also observed grown on copper sulfate containing agar plates. Applying 100 μM CuSO_4_ caused approximately 16% (± 4.5%), 1 mM CuSO_4_ approximately 69% (± 1.5%) decrease in colony diameter compared to normal growth conditions. This reduction in growth was only 3% (± 3%) in the WT ATCC46645 when grown on 100 μM CuSO_4_ plates and 50% (± 0%) and when 1 mM CuSO_4_ was present (**Figure 6B**). The increased sensitivity of the Δ*yap1* strain for copper and cadmium ions suggests that AfYap1 takes part in sensing these metal ions.

### PcaA Contributes to Virulence of *A. fumigatus* in the *Galleria mellonella* Infection Model

**FIGURE 7:**
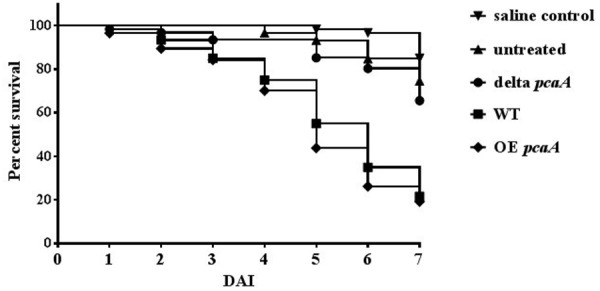
Virulence of the Δ*pcaA* and the OE *pcaA* strains using the *Galleria mellonella* infection model. The survival rate of the larvae infected with Δ*pcaA*, OE *pcaA* and the WT strain was measured daily for 7 days after infection (DAI). The data of three independent measurements are depicted, where groups of 12–28 larvae/strain were infected with 8 × 10^4^ conidiospores in 20 μl volume. The higher survival rate of the Δ*pcaA* indicates the decreased virulence of the deletion strain. The log- rank (Mantel–Cox) test was used for statistics calculated by the GraphPad Prism 7.00 software. The WT and Δ*pcaA* survival curves were significantly different (*P* < 0.0001).

Metal -including copper- homeostasis, is a crucial element in the virulence of pathogenic species (Festa and Thiele, 2012; Ding et al., 2014). Assuming common regulation in cadmium and copper homeostasis, the role of PcaA in virulence of the human pathogen *A. fumigatus* was tested. The virulence assay was performed using the *G. mellonella* (greater wax moth) infection model. Since the immune responses of this organism share similarities with the innate vertebrate immune response, the greater wax moth is a popular organism to investigate the virulence of microbial pathogens (Fallon et al., 2011; Slater et al., 2011; Tsai et al., 2016). Groups of 12–28 larvae were infected with the Δ*pcaA*, OE *pcaA* and the WT strains and survival of the infected larvae was monitored daily over a week. The survival rate of the larvae infected with the Δ*pcaA* mutant strain was significantly higher than of those which were infected with the WT strain (**Figure 7**). Consistently, the survival rate of the OE *pcaA* was slightly decreased in comparison to WT, showing that PcaA contributes to the virulence of *A. fumigatus*.

### The Excess of PcaA Results in Increased ROS Tolerance

**FIGURE 8:**
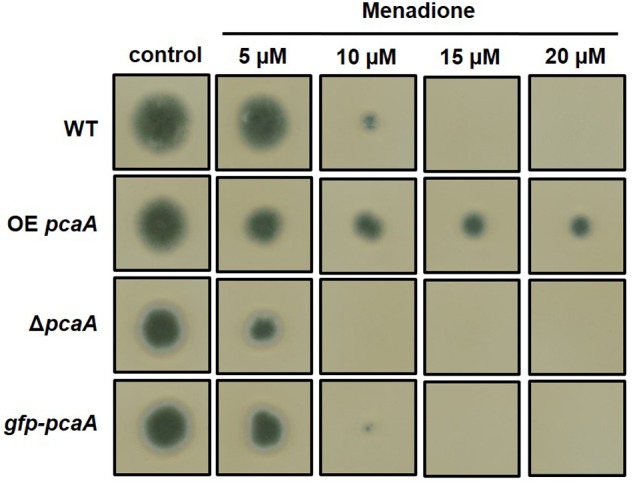
Growth of Δ*pcaA*, OE *pcaA*, and the *gfp-pcaA* complementation strains on menadione sodium bisulfite (MSB) containing agar plates. 3000 conidiospores/strain were point inoculated on agar plates and grown for 3 days at 37°C. Whereas applying lower concentrations of MSB inhibited the growth of Δ*pcaA* (see 10 μM), but not the WT and the *gfp-pcaA* complementation strain, the OE *pcaA* strain was still able to propagate colony on agar plates with high MSB concentrations (see 20 μM).

Based on the finding, that PcaA seemed to play a role in the virulence of *A. fumigatus*, the question was raised whether there is a correlation between virulence and the ROS tolerance, because the well-known defense strategy of host organisms is the production of ROS to combat the pathogen. Therefore, plate assays were carried out on medium containing oxidative stress generating drugs. We could show that increased PcaA protein levels resulted in elevated tolerance to menadione, whereas the lack of PcaA caused increased menadione sensitivity (**Figure 8**). This result supports the involvement of PcaA in ROS detoxification and maybe by mediating oxidative stress tolerance, supports the virulence of *A. fumigatus* in the *G. mellonella* model (**Figure 7**).

## Discussion

The subcellular localization and function of *A. fumigatus* PcaA -carrying the conserved features of a Pca1p -type transmembrane protein- was analyzed with a focus on heavy metal susceptibility and virulence.

### PcaA Is Localized to the Plasma Membrane of *A. fumigatus*

Fluorescence microscopy of the *gfp-pcaA* complementation strain was executed in the presence of different metals to follow the localization of the GFP-PcaA fusion protein. Under cadmium exposure, the GFP-PcaA fusion protein is accumulated in the plasma membrane, whereas no signal was detected in the presence of copper, zinc, or iron. This observation supports the *in silico* predictions of PcaA as a plasma membrane protein (**Figure 1**) and that cadmium is necessary to induce PcaA. GFP-PcaA was mainly found at the plasma membrane and in the plasma membrane covering the septae (**Figure 3C**). The septal presence of the GFP-PcaA might promote a defense mechanism to detoxify cadmium by excreting it to the next hyphal compartment. A septum-directed exocytosis through the septal cell wall of *Aspergillus oryzae* had been described (Hayakawa et al., 2011) and selective transport through the septal wall was shown in *Aspergillus niger* (Bleichrodt et al., 2015).

### Cadmium and Copper Ions Can Induce *pcaA* Transcription

Cadmium or copper ions are required to induce the transcription of *A. fumigatus* *pcaA* (**Figure 4**), which is reminiscent to the situation in the unicellular fungus *S. cerevisiae*, where copper and cadmium ions induce the transcription of the ortholog *pca1p* gene for copper resistance and cadmium detoxification (Adle et al., 2007). In *A. fumigatus*, the formation of detectable levels of PcaA protein was restricted to the induction by cadmium ions (**Figures 3B,C**), whereas copper ions only induced transcription without resulting in detectable protein (Supplementary Figure S4). Cadmium ions are necessary to stabilize the Pca1p protein of *S. cerevisiae* against 26S proteasome mediated degradation (Adle and Lee, 2008) and might fulfill a similar function in *A. fumigatus*. Worth to mention that Pca1p is inducible by copper conferring copper resistance to the baker’s yeast, albeit this resistance lies rather on the metal binding capacity of the N- terminal cysteines, than on ATPase activity (Adle et al., 2007). Although the N- terminus of *A. fumigatus* PcaA also carries a cysteine rich region (up to 8.2% cysteines in the N- terminal region) that might be involved in copper binding and through this, a transcriptional (up)regulation, yet, PcaA appeared to be specific for cadmium adaption (**Figure 2**). The induction of the copper exporter *crpA* by copper was recently shown (Wiemann et al., 2017). Here, we demonstrated the activation of the copper exporter encoding gene *crpA* by cadmium in the filamentous fungus *A. fumigatus* (**Figure 5A**). Different *crpA* expression patterns were observed in the presence or absence of PcaA under cadmium ion exposure (**Figure 5A**), whereas the gene expression of the copper specific protein CrpA was independent of PcaA when exposed to higher concentrations of copper ions (**Figure 5B**). The expression of genes for the metal exporters PcaA and CrpA is presumably a result of the early, more general and rapid response to cadmium stress, which response could be under the control of a mutual, yet elusive regulatory protein. Our data support an interconnection in the regulation of the transcriptome of copper and cadmium ion transport and homeostasis under specific stress conditions, e.g., cadmium when cells need a rapid response. The presence of a common metal sensing regulator can be suspected based on the chemical similarities of cadmium and copper ions. In yeast Yap1 and Yap2 -also known as CAD1- are transcription factors responsible for metal sensing and mediating pleiotropic drug- and metal resistance (Wu et al., 1993; Azevedo et al., 2007). Yap1 and Yap2 possess a cysteine- rich cadmium sensing domain located at the C-terminus (cCRD), which provides elevated cadmium tolerance when either Yap1 or Yap2 is overexpressed (Wu et al., 1993; Azevedo et al., 2007). The ortholog of the yeast Yap1 in *A. fumigatus* is AfYap1, responsible for oxidative stress response (Lessing et al., 2007; Qiao et al., 2008). AfYap1, similarly to yeast Yap1 and Yap2, contains a C-terminal cysteine rich region (Cys419; Cys431; Cys438; Cys562; Cys586; Cys595). The CRD region of AfYap1 presumably takes part in sensing and/or binding the intracellular thiol-reactive metals as cadmium and copper. The decreased cadmium and copper ion tolerance of the *Afyap1* deletion strain supports the presumption that AfYap1 takes part in heavy metal ion sensing (**Figure 6**), although the decreased resistance in the AfYap1 deficient strain might be due to oxidative stress caused by cadmium or copper ions.

### PcaA Contributes to the Virulence of *A. fumigatus* in the *G. mellonella* Infection Model

Metal, including copper homeostasis is a crucial process in the virulence of pathogenic species (Festa and Thiele, 2012; Ding et al., 2014). Acquisition, intracellular transport and excretion of copper can promote the virulence of the gram- negative bacterium *E. coli* as well as the fungal dimorphic pathogens *Cryptococcus neoformans* or *C. albicans* (Weissman et al., 2000; White et al., 2009; Waterman et al., 2012; Zhang et al., 2016). A link between the function of PcaA and virulence was examined in the *G. mellonella* infection model, as the immune responses of this organism share similarities with the response of the innate vertebrate immune system (Fallon et al., 2011; Tsai et al., 2016). Lack of PcaA remarkably attenuated the virulence of *A. fumigatus* in the wax moth larvae, suggesting the contribution of PcaA in virulence (**Figure 7**). Gene expression measurements suggest a connection in the fine-tuned transcription regulation of PcaA in the CrpA genes (**Figure 5A**) leading us to the assumption that the lack of PcaA might influence the induction of other proteins which may be involved in virulence through metal transport or homeostasis. CrpA is involved in copper detoxification by extrusion of copper ions, as well as in the virulence of *A. fumigatus* (Wiemann et al., 2017). A connection of oxidative stress defense and copper metabolism had been reported and both promote virulence of pathogenic species (Wiemann et al., 2017). Similarly, the excess of PcaA resulted in elevated, the lack of PcaA resulted in decreased oxidative stress tolerance against the superoxide generating agent menadione (**Figure 8**). The elevated oxidative stress tolerance of the OE *pcaA* strain might be mediated by the N- terminal cysteine rich region of PcaA. The first N- terminal 400 amino acid sequence of PcaA contains 8.2% cysteines, which is the third most abundant amino acid of this region [calculated with ProtParam, ExPasy (Gasteiger et al., 2003)]. In yeast, the metallothionein CUP1 has an antioxidant effect and can partially restore the phenotypes caused by the deletion of the superoxide dismutase SOD1 (Tamai et al., 1993). PcaA might be able to eliminate superoxide radicals generated by menadione through its N-terminal cysteine rich region.

The involvement of ATPases in oxidative stress tolerance and virulence is not yet fully understood in *A. fumigatus*, hence to corroborate these hypotheses, further research is required. Promising, that so far no orthologous cadmium exporting ATPase was described in humans, thus, analysis of PcaA -as a fungal specific protein and putative target for antifungal drugs- might bring us closer to combat this important opportunistic human pathogen.

## Author Contributions

IP initiated the project. FB planned and carried out the experiments. FB, CS, and GB interpreted the data and wrote the manuscript. TH analyzed the ΔAf*yap1* strain.

## Conflict of Interest Statement

The authors declare that the research was conducted in the absence of any commercial or financial relationships that could be construed as a potential conflict of interest.

## References

[B1] Adle D. J. Lee J. (2008). Expressional control of a cadmium-transporting P1B-type ATPase by a metal sensing degradation signal. *J. Biol. Chem.* 283 31460–31468. 10.1074/jbc.M806054200 18753133 PMC2581566

[B2] Adle D. J. Sinani D. Kim H. Lee J. (2007). A cadmium-transporting P1B-type ATPase in yeast *Saccharomyces cerevisiae*. *J. Biol. Chem.* 282 947–955. 10.1074/jbc.M609535200 17107946 PMC4100611

[B3] Aguirre J. Hansberg W. Navarro R. (2006). Fungal responses to reactive oxygen species. *Med. Mycol.* 44 S101–S107. 10.1080/13693780600900080 30408892

[B4] Azevedo D. Nascimento L. Labarre J. Toledano M. B. Rodrigues-Pousada C. (2007). The *S. cerevisiae* Yap1 and Yap2 transcription factors share a common cadmium-sensing domain. *FEBS Lett.* 581 187–195. 10.1016/j.febslet.2006.11.083 17187783

[B5] Bertin G. Averbeck D. (2006). Cadmium: cellular effects, modifications of biomolecules, modulation of DNA repair and genotoxic consequences (a review). *Biochimie* 88 1549–1559. 10.1016/j.biochi.2006.10.001 17070979

[B6] Bhabhra R. Askew D. S. (2005). Thermotolerance and virulence of *Aspergillus fumigatus*: role of the fungal nucleolus. *Med. Mycol.* 43 S87–S93. 16110798 10.1080/13693780400029486

[B7] Bleichrodt R.-J. Vinck A. Read N. D. Wösten H. A. B. (2015). Selective transport between heterogeneous hyphal compartments via the plasma membrane lining septal walls of *Aspergillus niger*. *Fungal Genet. Biol.* 82 193–200. 10.1016/j.fgb.2015.06.010 26212073

[B8] Borkow G. Gabbay J. (2009). Copper, an ancient remedy returning to fight microbial, fungal and viral infections. *Curr. Chem. Biol.* 3 272–278. 10.2174/187231309789054887

[B9] Brown N. A. Goldman G. H. (2016). The contribution of *Aspergillus fumigatus* stress responses to virulence and antifungal resistance. *J. Microbiol.* 54 243–253. 10.1007/s12275-016-5510-4 26920884

[B10] De Vries R. P. Riley R. Wiebenga A. Aguilar-Osorio G. Amillis S. Uchima C. A. (2017). Comparative genomics reveals high biological diversity and specific adaptations in the industrially and medically important fungal genus Aspergillus. *Genome Biol.* 18:28. 10.1186/s13059-017-1151-0 28196534 PMC5307856

[B11] Ding C. Festa R. A. Sun T. S. Wang Z. Y. (2014). Iron and copper as virulence modulators in human fungal pathogens. *Mol. Microbiol.* 93 10–23. 10.1111/mmi.12653 24851950

[B12] Djoko K. Y. Ong C. Y. Walker M. J. McEwan A. G. (2015). The role of copper and zinc toxicity in innate immune defense against bacterial pathogens. *J. Biol. Chem.* 290 18954–18961. 10.1074/jbc.R115.647099 26055706 PMC4521016

[B13] Fallon J. P. Troy N. Kavanagh K. (2011). Pre-exposure of *Galleria mellonella* larvae to different doses of *Aspergillus fumigatus* conidia causes differential activation of cellular and humoral immune responses. *Virulence* 2 413–421. 10.4161/viru.2.5.17811 21921688

[B14] Fazli M. M. Soleimani N. Mehrasbi M. Darabian S. Mohammadi J. Ramazani A. (2015). Highly cadmium tolerant fungi: their tolerance and removal potential. *J. Environ. Health Sci. Eng.* 13:19. 10.1186/s40201-015-0176-0 25806110 PMC4372280

[B15] Festa R. A. Thiele D. J. (2011). Copper: an essential metal in biology. *Curr. Biol.* 21 R877–R883. 10.1016/j.cub.2011.09.040 22075424 PMC3718004

[B16] Festa R. A. Thiele D. J. (2012). Copper at the front line of the host-pathogen battle. *PLoS Pathog.* 8:e1002887. 10.1371/journal.ppat.1002887 23028306 PMC3447745

[B17] Gaetke L. M. Chow C. K. (2003). Copper toxicity, oxidative stress, and antioxidant nutrients. *Toxicology* 189 147–163. 10.1016/S0300-483X(03)00159-8 12821289

[B18] Gasteiger E. Gattiker A. Hoogland C. Ivanyi I. Appel R. D. Bairoch A. (2003). ExPASy: the proteomics server for in-depth protein knowledge and analysis. *Nucleic Acids Res.* 31 3784–3788. 10.1093/nar/gkg563 12824418 PMC168970

[B19] Gomez-Lopez A. Forastiero A. Cendejas-Bueno E. Gregson L. Mellado E. Howard S. J. (2014). An invertebrate model to evaluate virulence in *Aspergillus fumigatus*: the role of azole resistance. *Med. Mycol.* 52 311–319. 10.1093/mmy/myt022 24577012

[B20] Hartmann T. Dümig M. Jaber B. M. Szewczyk E. Olbermann P. Morschhäuser J. (2010). Validation of a self-excising marker in the human pathogen *Aspergillus fumigatus* by employing the βrec/six site-specific recombination system. *Appl. Environ. Microbiol.* 76 6313–6317. 10.1128/AEM.00882-10 20656854 PMC2937505

[B21] Hayakawa Y. Ishikawa E. Shoji J.-Y. Nakano H. Kitamoto K. (2011). Septum-directed secretion in the filamentous fungus *Aspergillus oryzae*. *Mol. Microbiol.* 81 40–55. 10.1111/j.1365-2958.2011.07700.x 21564341

[B22] Hwang C.-S. Rhie G.-E. Oh J.-H. Huh W.-K. Yim H.-S. Kang S.-O. (2017). Copper-and zinc-containing superoxide dismutase (Cu/ZnSOD) is required for the protection of *Candida albicans* against oxidative stresses and the expression of its full virulence. *Microbiology* 1027 3705–3713. 12427960 10.1099/00221287-148-11-3705

[B23] Inoue H. Nojima H. Okayama H. (1990). High efficiency transformation of *Escherichia coli* with plasmids. *Gene* 96 23–28. 10.1016/0378-1119(90)90336-P 2265755

[B24] Jesenská Z. Piecková E. Bernát D. (1993). Heat resistance of fungi from soil. *Int. J. Food Microbiol.* 19 187–192. 10.1016/0168-1605(93)90076-S 8217516

[B25] Jöhnk B. Bayram O. Abelmann A. Heinekamp T. Mattern D. J. Brakhage A. A. (2016). SCF ubiquitin ligase F-box protein Fbx15 controls nuclear co-repressor localization, stress response and virulence of the human pathogen *Aspergillus fumigatus*. *PLoS Pathog.* 12:e1005899. 10.1371/journal.ppat.1005899 27649508 PMC5029927

[B26] Käfer E. (1977). Meiotic and mitotic recombination in Aspergillus and its chromosomal aberrations. *Adv. Genet.* 19 33–131. 10.1016/S0065-2660(08)60245-X 327767

[B27] Kelley L. A. Mezulis S. Yates C. M. Wass M. N. Sternberg M. J. E. (2015). The Phyre2 web portal for protein modelling, prediction and analysis. *Nat. Protoc.* 10 845–858. 10.1038/nprot.2015.053 25950237 PMC5298202

[B28] Kühlbrandt W. (2004). Biology, structure and mechanism of P-type ATPases. *Nat. Rev. Mol. Cell Biol.* 5 282–295. 10.1038/nrm1354 15071553

[B29] Lessing F. Kniemeyer O. Wozniok I. Loeffler J. Kurzai O. Haertl A. (2007). The *Aspergillus fumigatus* transcriptional regulator AfYap1 represents the major regulator for defense against reactive oxygen intermediates but is dispensable for pathogenicity in an intranasal mouse infection model. *Eukaryot. Cell* 6 2290–2302. 10.1128/EC.00267-07 17921349 PMC2168236

[B30] Livak K. J. Schmittgen T. D. (2001). Analysis of relative gene expression data using real-time quantitative PCR and the ΔΔCT method. *Methods* 25 402–408. 10.1006/meth.2001.1262 11846609

[B31] Mackie J. Szabo E. K. Urgast D. S. Ballou E. R. Childers D. S. Maccallum D. M. (2016). Host-imposed copper poisoning impacts fungal micronutrient acquisition during systemic *Candida albicans* infections. *PLoS ONE* 11:e0158683. 10.1371/journal.pone.0158683 27362522 PMC4928837

[B32] McMurray C. T. Tainer J. A. (2003). Cancer, cadmium and genome integrity. *Nat. Genet.* 34 239–241. 10.1038/ng0703-239 12833042

[B33] Muszkieta L. Carrion S. de J. Robinet P. Beau R. Elbim C. (2014). The protein phosphatase PhzA of *A. fumigatus* is involved in oxidative stress tolerance and fungal virulence. *Fungal Genet. Biol.* 66 79–85. 10.1016/j.fgb.2014.02.009 24614084 PMC4503245

[B34] Pontecorvo G. Roper J. A. Hemmons L. M. Macdonald K. D. Bufton A. W. (1953). The genetics of *Aspergillus nidulans*. *Adv. Genet.* 5 141–238. 10.1016/S0065-2660(08)60408-3 13040135

[B35] Punt P. J. van den Hondel C. A. M. J. J. (1992). Transformation of filamentous fungi based on hygromycin B and phleomycin resistance markers. *Methods Enzym.* 216 447–457. 10.1016/0076-6879(92)16041-H 1479914

[B36] Qiao J. Kontoyiannis D. P. Calderone R. Li D. Ma Y. Wan Z. (2008). Afyap1, encoding a bZip transcriptional factor of *Aspergillus fumigatus*, contributes to oxidative stress response but is not essential to the virulence of this pathogen in mice immunosuppressed by cyclophosphamide and triamcinolone. *Med. Mycol.* 46 773–782. 10.1080/13693780802054215 18608886

[B37] Riggle P. J. Kumamoto C. A. (2000). Role of a *Candida albicans* P1-type ATPase in resistance to copper and silver ion toxicity. *J. Bacteriol.* 182 4899–4905. 10.1128/JB.182.17.4899-4905.2000 10940034 PMC111370

[B38] Shlezinger N. Irmer H. Dhingra S. Beattie S. R. Cramer R. A. Braus G. H. (2017). Sterilizing immunity in the lung relies on targeting fungal apoptosis-like programmed cell death. *Science* 357 1037–1041. 10.1126/science.aan0365 28883073 PMC5628051

[B39] Slater J. L. Gregson L. Denning D. W. Warn P. A. (2011). Pathogenicity of *Aspergillus fumigatus* mutants assessed in *Galleria mellonella* matches that in mice. *Med. Mycol.* 49 S107–S113. 10.3109/13693786.2010.523852 20950221

[B40] Stafford S. L. Bokil N. J. Achard M. E. S. Kapetanovic R. Schembri M. A. Mcewan A. G. (2013). Metal ions in macrophage antimicrobial pathways: emerging roles for zinc and copper. *Biosci. Rep.* 33:e00049. 10.1042/BSR20130014 23738776 PMC3712485

[B41] Tamai K. T. Gralla E. B. Ellerby L. M. Valentine J. S. Thiele D. J. (1993). Yeast and mammalian metallothioneins functionally substitute for yeast copper-zinc superoxide dismutase. *Biochemistry* 90 8013–8017. 10.1073/pnas.90.17.8013 8367458 PMC47278

[B42] Tekaia F. Latgé J.-P. (2005). *Aspergillus fumigatus*: saprophyte or pathogen? *Curr. Opin. Microbiol.* 8 385–392. 10.1016/j.mib.2005.06.017 16019255

[B43] Tsai C. J.-Y. Loh J. M. S. Proft T. (2016). *Galleria mellonella* infection models for the study of bacterial diseases and for antimicrobial drug testing. *Virulence* 7 214–229. 10.1080/21505594.2015.1135289 26730990 PMC4871635

[B44] Upadhyay S. Torres G. Lin X. (2013). Laccases involved in 1,8-dihydroxynaphthalene melanin biosynthesis in *Aspergillus fumigatus* are regulated by developmental factors and copper homeostasis. *Eukaryot. Cell* 12 1641–1652. 10.1128/EC.00217-13 24123270 PMC3889567

[B45] Wagener J. Echtenacher B. Rohde M. Kotz A. Krappmann S. Heesemann J. (2008). The putative alpha-1,2-mannosyltransferase AfMnt1 of the opportunistic fungal pathogen *Aspergillus fumigatus* is required for cell wall stability and full virulence. *Eukaryot. Cell* 7 1661–1673. 10.1128/EC.00221-08 18708564 PMC2568062

[B46] Waterman S. R. Park Y.-D. Raja M. Qiu J. Hammoud D. A. O’halloran T. V. (2012). Role of CTR4 in the virulence of *Cryptococcus neoformans*. *MBio* 3:e00285-12. 10.1128/mBio.00285-12 23033470 PMC3518914

[B47] Weissman Z. Berdicevsky I. Cavari B.-Z. Kornitzer D. (2000). The high copper tolerance of *Candida albicans* is mediated by a P-type ATPase. *Proc. Natl. Acad. Sci. U.S.A.* 97 3520–3525. 10.1073/pnas.97.7.3520 10737803 PMC16272

[B48] White C. Lee J. Kambe T. Fritsche K. Petris M. J. (2009). A role for the ATP7A copper-transporting ATPase in macrophage bactericidal activity. *J. Biol. Chem.* 284 33949–33956. 10.1074/jbc.M109.070201 19808669 PMC2797165

[B49] Wiederhold N. Patterson T. (2015). Emergence of azole resistance in Aspergillus. *Semin. Respir. Crit. Care Med.* 36 673–680. 10.1055/s-0035-1562894 26398534

[B50] Wiemann P. Perevitsky A. Huttenlocher A. Osherov N. Keller N. P. (2017). *Aspergillus fumigatus* copper export machinery and reactive oxygen intermediate defense counter host copper-mediated oxidative antimicrobial offense. *Cell Rep.* 19 1008–1021. 10.1016/j.celrep.2017.04.019 28467895 PMC5512462

[B51] Wu A. Wemmie J. A. Edgington N. P. Goebl M. Guevara J. L. Moye-Rowley W. S. (1993). Yeast bZip proteins mediate pleiotropic drug and metal resistance. *J. Biol. Chem.* 288 18850–18858. 8360174

[B52] Zhang P. Zhang D. Zhao X. Wei D. Wang Y. Zhu X. (2016). Effects of CTR4 deletion on virulence and stress response in *Cryptococcus neoformans*. *Antonie Van Leeuwenhoek* 109 1081–1090. 10.1007/s10482-016-0709-2 27317510

