# Supplementary material for: Heavy Metal-Induced Expression of PcaA Provides Cadmium Tolerance to Aspergillus fumigatus and Supports Its Virulence in the Galleria mellonella Model
Source: Front Microbiol. 2018 Apr 13;9:744. doi: 10.3389/fmicb.2018.00744 (PMC5909057; doi:10.3389/fmicb.2018.00744)
Supplement: Supplementary file 1 [file Data_Sheet_1.pdf]

*Supplementary Material*

# Heavy Metal Induced Expression of PcaA Provides Cadmium Tolerance to *Aspergillus fumigatus* and Supports its Virulence in the *Galleria mellonella* Model

**Fruzsina Bakti[1,3], Christoph Sasse[1], Thorsten Heinekamp[2], István Pócsi[3] and Gerhard H. Braus[1*]**

[1]Institute for Microbiology and Genetics, Department of Molecular Microbiology and Genetics, and Goettingen Center for Molecular Biosciences (GZMB), University of Goettingen, Goettingen, Germany

[2]Department of Molecular and Applied Microbiology Leibniz Institute for Natural Product Research and Infection Biology - Hans-Knoell-Institute (HKI), Jena, Germany

[3]Department of Biotechnology and Microbiology, Faculty of Science and Technology, University of Debrecen, Debrecen, Hungary

[*] E-mail: gbraus@gwdg.de

## 1 Supplementary Tables

**Table S1.** ***Aspergillus fumigatus*** **strains of this study.**

| Strain | Description | Reference |
|---|---|---|
| Af293 | wild type | Fungal Genetics Stock Center, Kansas City, Missouri USA |
| OE *pcaA* | $^{P}$*gpdA::pcaA::ptrA*$^{R}$ | This study |
| Δ*pcaA* | Δ*pcaA:six* | This study |
| *gfp-pcaA* | Δ*pcaA:six::gfp-pcaA:six* | This study |
| Δ*Afyap1* | *Afyap1::hph*; Hygr$^{R}$ | (Lessing et al., 2007) |
| *yap1*$^{compl}$ | Δ*Afyap1::Afyap1* | (Lessing et al., 2007) |
| ATCC46645 | wild type | (Lessing et al., 2007) |

**Table S2. Interspecies comparison with the deduced amino acid sequence of PcaA.** BLASTp search was carried out to compare deduced amino acid sequence of the listed species. The table represents the amino acid identities of the full-length protein and the HMA domains of the regarding proteins.

| Systematic Name | UniProt ID | Protein Name | Species | Protein | | HMA domain | | |
|---|---|---|---|---|---|---|---|---|
| | | | | Identity | E-value | Location (aa) | Identity | E-value |
| Afu1g16130 | Q4WRJ0 | PcaA | *A. fumigatus* | - | - | 320-378 | - | - |
| YBR295W | P38360 | Pca1p | *S. cerevisiae* | 51 % | 0.0 | 411-474 | 57 % | 1e-27 |
| C1_09250W_A | Q9UVL6 | CRP1 | *C. albicans* | 28 % | 5e -75 | 171-242 | 30 % | 7e-04 |
| | | | | | | 255-322 | 29 % | 1e-06 |
| Afu4g12620 | Q4WQF3 | CtpA | *A. fumigatus* | 29.6 % | 2e-87 | 25-91 | 29 % | 1e-08 |
| | | | | | | 116-182 | 30 % | 5e-08 |
| | | | | | | 207-273 | 32 % | 3e-08 |
| Afu3g12740 | Q4WYE4 | CrpA | *A. fumigatus* | 26.4 % | 1e-65 | 205-271 | 26 % | 5e-06 |
| | | | | | | 288-353 | 31 % | 1e-07 |

**Table S3. Primers used in this study.**

| Name | Size (bp) | Sequence (5'→ 3') |
|---|---|---|
| pca1-1 | 40 | CTG CAG GAA TTC GAT GTT TAA ACT GGG TAT GTG GGT AGA G |
| pca1-2 | 41 | ACC TAT AGG CCT GAG CAC GTG CGT TGA AGT CAG CGC TTG CT |
| pca1-3 | 35 | ATA ATA TGG CCA TCT CCA CAG AGC ACC GTT ATT GC |
| pca1-4 | 46 | ATC GAT AAG CTT GAT GTT TAA ACT TTA GAA CAT TAT TTT AAA TCT T |
| FB008 | 35 | ATA GAC ATG GCG TTT CTA GAT CTT CGA CCA GCG CA |
| FB009 | 35 | CAG ACA TCA CCG TTT ATG GGA GAC GAC TAT TGC GG |
| FB033 | 35 | GCC CTT GCT CAC CAT CGT TGA AGT CAG CGC TTG CT |
| FB034 | 40 | ACA AGG GTG GTA GCG GTG GTA TGG GAG ACG ACT ATT GCG G |
| FB075 | 42 | CTG CAG GAA TTC GAT GTT TAA ACT GGG TAT GTG GGT AGA GGA |
| FB077 | 33 | ATA ATA TGG CCA TCT CCA CAG AGC ACC GTT ATT |

| | | |
|---|---|---|
| FB078 | 43 | ATC GAT AAG CTT GAT ATT TAA ATC TAG ATC TTC GAC CAG CGC A |
| FB084 | 46 | ATA AGC TTG ATA TTT GTT TAA ACT TTA GAA CAT TAT TTT AAA TCT T |
| FB094 | 22 | GAA TGC GAG ATG AGT GCT ACA G |
| FB095 | 22 | GAG GGC AGA GAG ACT ATG TTG G |
| FB104 | 20 | GCG AGA TGG TCA CCG TTA AT |
| FB105 | 20 | ACC GAC CCA ACA GTT TCT TG |
| SR120 | 21 | ATG GTG AGC AAG GGC GAG GAG |
| SR121 | 36 | ACC ACC GCT ACC ACC CTT GTA CAG TTC GTC CAT GCC |
| KT316 | 20 | TGG AGT ATC TCG CTG CTG AA |
| KT317 | 20 | GGA GAT GGC GAG GAA TGA TA |

## Table S4. Plasmid constructs used in this study.

| Name | Description | Parental plasmid | Reference |
|---|---|---|---|
| pBluescript II KS+ | cloning vector; amp$^{R}$ | | Fermentas |
| pSK485 | *ptrA* recyclable marker cassette | | (Hartmann et al., 2010) |
| pSK379 | $^{p}$*gpdA-ptrA*$^{R}$*-his2A*$^{t}$; amp$^{R}$ | | (Wagener et al., 2008) |
| pME4292 | GFP in the *Mss*I site of pSK379 | pSK379 | (Jöhnk et al., 2016) |
| pFB03 | 5' UTR *pcaA*-six-$^{p}$xyl-β-rec-*trpC*$^{t}$-*prtA*$^{R}$-six-3'UTR *pcaA*; amp$^{R}$ | pBluescript II KS+ | This study |
| pFB08 | $^{P}$*gpdA-ptrA*$^{R}$*-pcaA* cds-*his2A*$^{t}$; amp$^{R}$ | pSK379 | This study |
| pFB22 | 5' UTR *pcaA*- sGFP- *pcaA*-six-$^{p}$xyl-β-rec-*trpC*$^{t}$-*prtA*$^{R}$-six-3' UTR *pcaA*; amp$^{R}$ | pBluescript II KS+ | This study |

## 2 Supplementary Figures

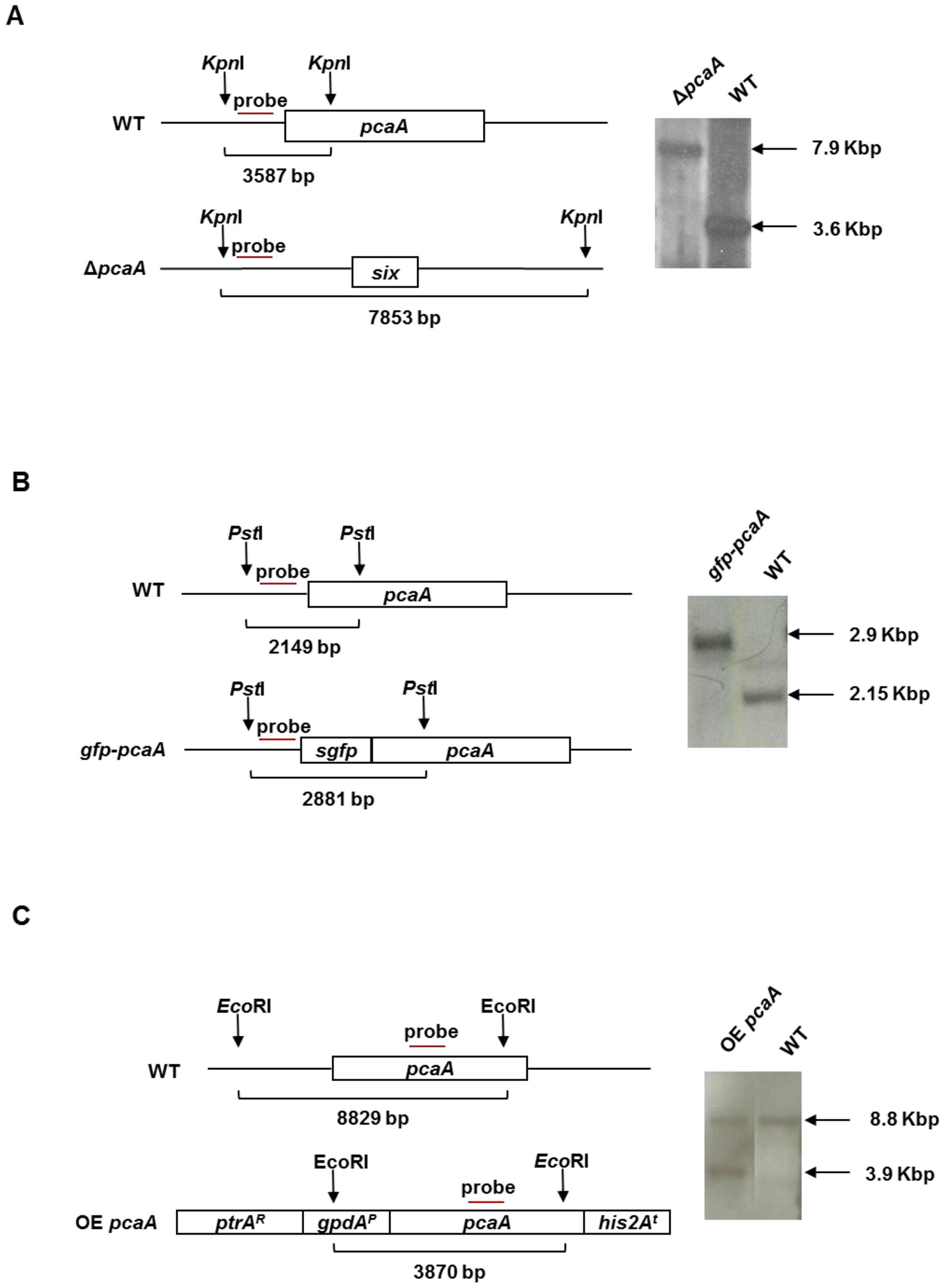

**Figure S1. Genetic manipulations and Southern hybridization analyses of *A. fumigatus* Δ*pcaA*, *gfp-pcaA* and OE *pcaA* strains. (A)** Deletion of *A. fumigatus pcaA* in the Af293 wild type background was performed using the recyclable marker system (Hartmann et al., 2010). After marker recycling the *pcaA* coding sequence is replaced by a 90 bp β-recombinase recognition site called *six*. **(B)** The *gfp-pcaA* strain was constructed in the Δ*pcaA* background strain, where the *gfp-pcaA* construct is integrated into the *pcaA* locus in a single copy, driven by its endogenous promoter. **(C)** The OE *pcaA* strain was created by the ectopic integration of the overexpression construct into the Af293 wild type strain. The *pcaA* gene expression is governed by the constitutive *gpdA* promoter.

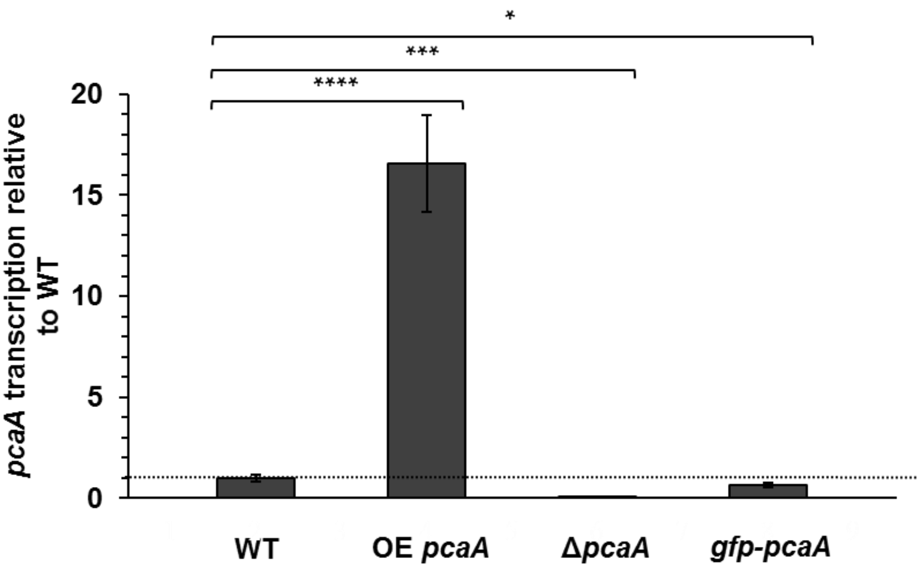


**Figure S2. The *pcaA* gene expression in the *ΔpcaA*, OE *pcaA* and the *gfp-pcaA* strains of *A. fumigatus*.** Samples of the above mentioned strains were collected from 20 hours old cultures. The charts represent the gene expression of *pcaA* in the indicated strains during vegetative growth in modified minimal medium without any supplementation. The error bars represent the standard error of the mean (SEM) of three technical replicates in two independent measurements. The asterisks indicate the significances between the control and the experimental sample (two-sided t-test, P<0.05 *; P<0.01 **; P<0.005 ***; P<0.001 ****). The dashed line indicates the expression of the control sample (wild type).

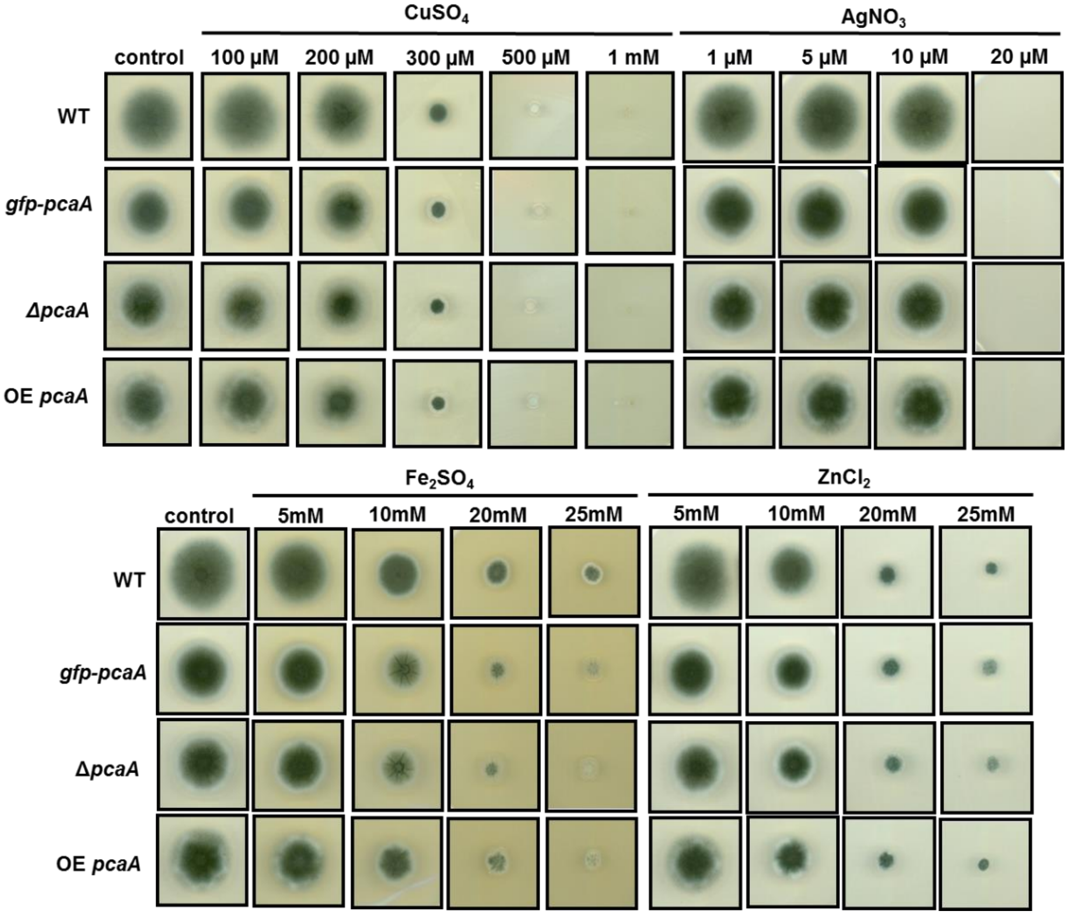


**Figure S3. Growth of Δ*pcaA*, OE *pcaA* and the *gfp-pcaA A. fumigatus* strains on metal containing agar plates.** Metal susceptibility of the above mentioned strains was tested on agar plates containing increasing concentrations of $CuSO_4$ ranging from 100 µM to 1 mM and $Fe_2SO_4$, $ZnCl_2$ ranging between 5 to 25 mM and $AgNO_3$ from 1 µM to 20 µM. 3000 conidiospores/strain were point inoculated on agar plates and grown for 3 days at 37°C. The phenotype of the examined strains did not show significant difference when they were grown on copper, iron, zinc or silver ion containing agar plates.

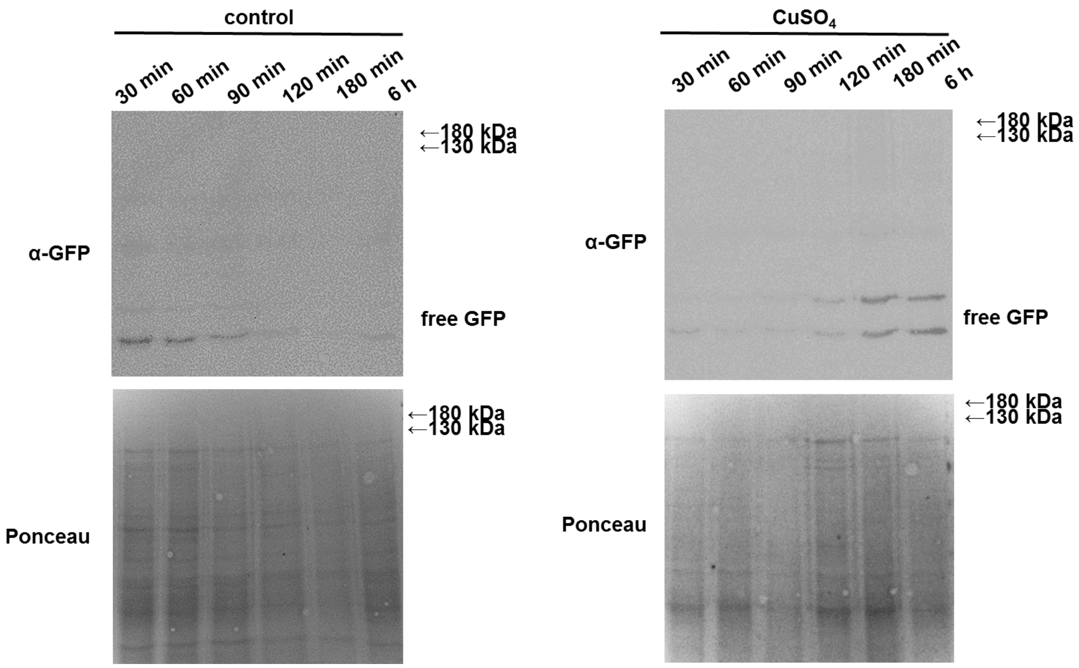


**Figure S4. The GFP-PcaA fusion protein is not detectable in fungal extracts in the presence of copper ions.** Immunoblotting was carried out with protein samples deriving from *gfp-pcaA* cultures with 300 µM copper sulfate and without supplementation. Samples were taken at the indicated time points following the supplementation. The western hybridization with a monoclonal α-GFP antibody showed no signal for the GFP-PcaA fusion protein (~149 kDa) in the control samples and not in the samples treated with copper sulfate, on the other hand, free GFP signal was detected indicating the degradation of GFP-PcaA.
